# Balancing Activity and Stability through Compositional Engineering of Ternary PtNi–Au Alloy ORR Catalysts

**DOI:** 10.1021/acscatal.4c05269

**Published:** 2024-12-16

**Authors:** Xianxian Xie, Valentín Briega-Martos, Pere Alemany, Athira Lekshmi Mohandas Sandhya, Tomáš Skála, Miquel Gamón Rodríguez, Jaroslava Nováková, Milan Dopita, Michael Vorochta, Albert Bruix, Serhiy Cherevko, Konstantin M. Neyman, Iva Matolínová, Ivan Khalakhan

**Affiliations:** † Department of Surface and Plasma Science, Faculty of Mathematics and Physics, Charles University, V Holešovičkách 2, 180 00 Prague 8, Czech Republic; ‡ Helmholtz Institute Erlangen-Nürnberg for Renewable Energy (IET-2), Forschungszentrum Julich GmbH, Cauerstr. 1, 91058 Erlangen, Germany; § Departament de Ciència de Materials i Química Física and Institut de Quimica Teòrica i Computacional (IQTCUB), Universitat de Barcelona, c/Martí i Franquès 1, 08028 Barcelona, Spain; ∥ Department of Condensed Matter Physics, Faculty of Mathematics and Physics, Charles University, 12116 Prague 2, Czech Republic; ⊥ ICREA (Institució Catalana de Recerca i Estudis Avançats), Pg. Lluís Companys 23, 08010 Barcelona, Spain

**Keywords:** fuel cells, oxygen reduction reaction, ternary alloy electrocatalyst, activity–stability relationship, optimal composition

## Abstract

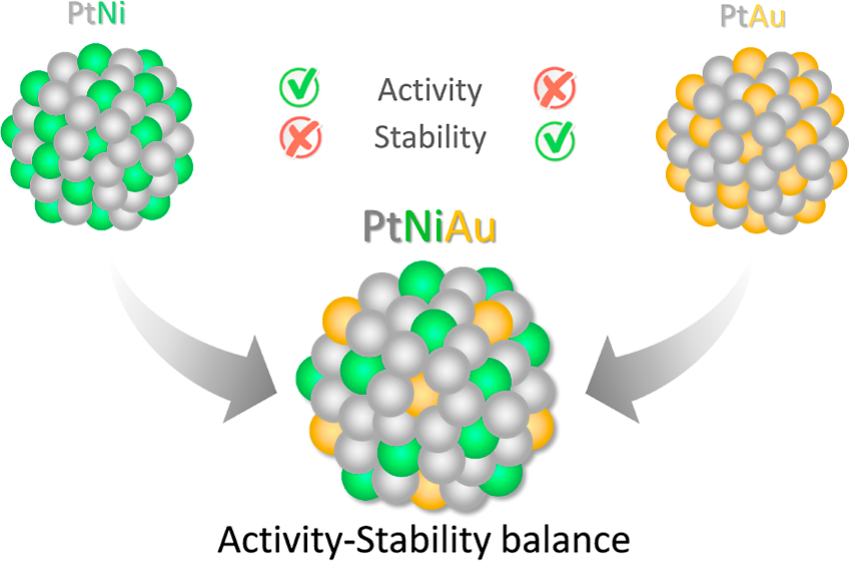

Achieving the optimal balance between cost-efficiency and stability of oxygen reduction reaction (ORR) catalysts is currently among the key research focuses aiming at reaching a broader implementation of proton-exchange membrane fuel cells (PEMFCs). To address this challenge, we combine two well-established strategies to enhance both activity and stability of platinum-based ORR catalysts. Specifically, we prepare ternary PtNi–Au alloys, where each alloying element plays a distinct role: Ni reduces costs and boosts ORR activity, while Au enhances stability. A systematic comparative analysis of the activity–stability relationship for compositionally tuned PtNi–Au model layers, prepared by magnetron co-sputtering, was conducted using a diverse range of complementary characterization techniques and electrochemistry, supported by density functional theory calculations. Our study reveals that a progressive increase of the Au concentration in the Pt_50_Ni_50_ alloy from 3 to 15 at % leads to opposing catalyst activity and stability trends. Specifically, we observe a decrease in the ORR activity accompanied by an increase in catalyst stability, manifested in the suppression of both Pt and Ni dissolution. Despite the reduced activity compared to PtNi, the PtNi–Au alloy with 15 at % Au still exhibits nearly three times the activity of monometallic Pt. It also demonstrates a significantly improved dissolution stability relative to that of the PtNi alloy and even monometallic Pt. These findings provide valuable insights into the intricate balance between activity and stability in multimetallic ORR catalysts, paving the way for the design of cost-effective and durable materials for PEMFCs.

## 1 Introduction

The pursuit for cost-efficient and stable catalysts for the oxygen reduction reaction (ORR) stands among the paramount quests for the broader implementation of advanced energy technologies, such as proton-exchange membrane fuel cells (PEMFCs). However, a significant challenge arises in the form of the so-called activity–stability trade-off. For instance, it has been well established that platinum alloyed with inexpensive 3d transition metals like nickel, cobalt, etc., markedly enhancing the catalyst cost-efficiency^1−3^ and significantly compromising its durability.^4−8^ On the contrary, the incorporation of gold into platinum has recently been proven as an effective strategy to stabilize the platinum catalyst.^9−11^ Yet, Pt–Au alloys do not demonstrate an enhancement in ORR activity nor a reduction in the overall catalyst cost.^10^ The delicate balance between activity and stability thus underscores the ongoing quest for optimal catalyst designs in fuel cell technology. Potentially, this could be achieved by combining the two approaches. Alloying of platinum with transition metal will reduce the catalyst cost-efficiency, while at the same time, the addition of Au will contribute to increased stability.

Indeed, ternary Pt–Au–M catalysts of various structure, composition, and shape have been tested as promising ORR catalysts, demonstrating increased activity and stability. For example, Kang et al. reported a Ni@Au@PtNi catalyst with a core–interlayer–shell structure containing 5 at % of Au, which exhibited high mass activity (MA) and less than 10% activity loss after an accelerated degradation test (ADT) consisting of 10,000 potential cycles between 0.6 and 1.1 V_RHE_, in comparison to almost 40% loss for the PtNi catalyst.^12^ This behavior was linked to the synergistic effect between subsurface Au stabilization and the altered electronic structure of surface Pt atoms due to its interaction with subsurface Ni atoms. Using a similar Pt–Ni–Au system containing 5.3 wt % of Au, Liu et al. showed that while exhibiting higher activity than commercial Pt/C, it also showed increased stability, manifested in just a 16.8% drop of its initial MA during 10,000 ADT cycles, compared to 59.1% and 78.9% for Pt/C and Pt–Ni/C, respectively.^13^ Recently, Gao et al. reported that PtNiAu nanowires with a composition of 3.01:1.00:0.11 exhibited only an 18.3% decrease in MA after 20,000 cycles of ADT, compared to a 53.5% decrease for PtNi nanowire catalysts.^14^ Gatalo et al. showed that PtCu_3_ doping with less than 1% of Au greatly improved its stability, leading to significantly reduced Pt and Cu dissolution.^15^ Enhanced activity and durability were also shown for Au@Co@PtCoAu multilayer structure and L1_0_-PtCoAu_0.1_/C catalyst.^16,17^

**Figure 1:**
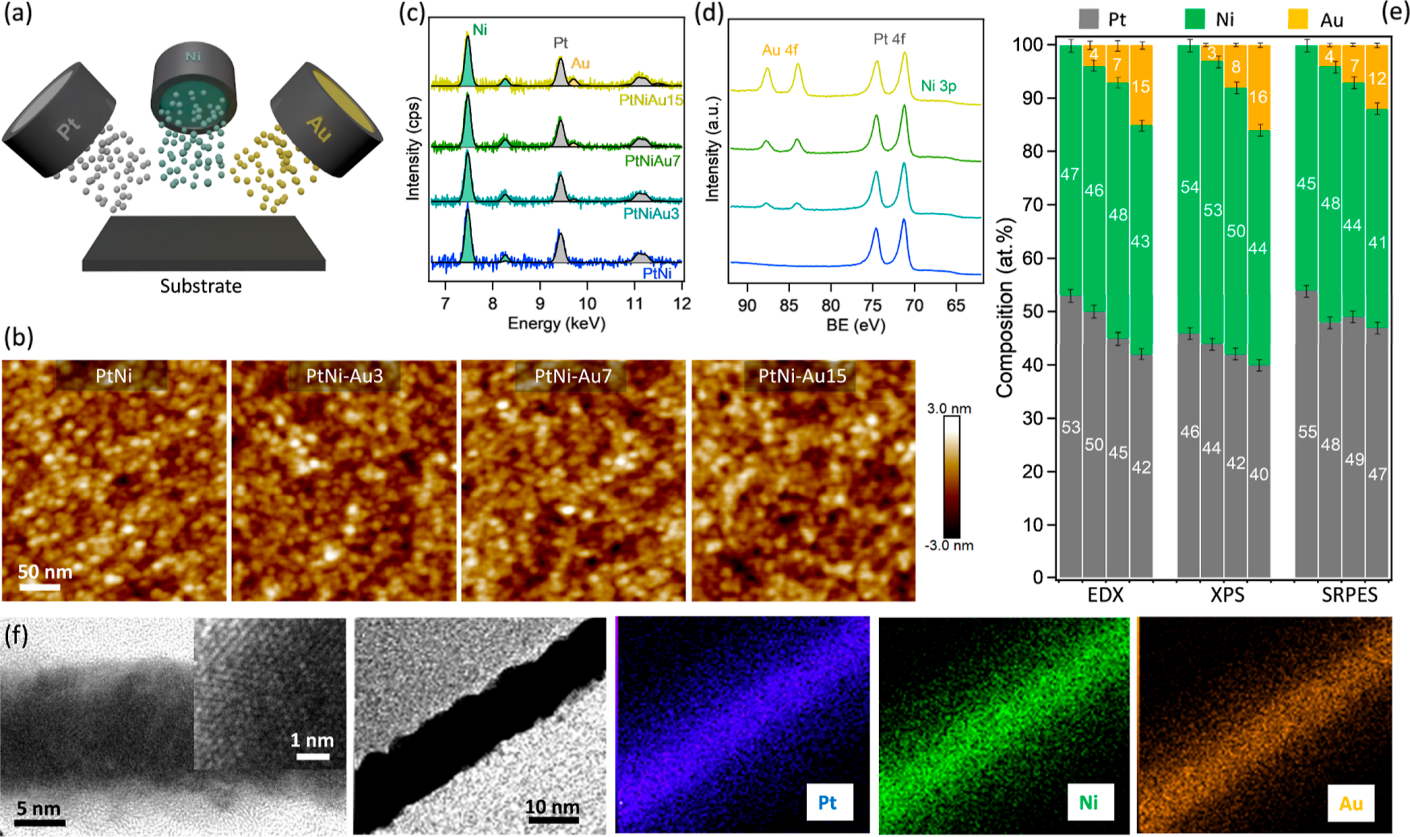
(a) Schematic illustration of magnetron co-sputtering deposition of ternary PtNi–Au catalysts; (b) AFM images of the as-deposited Pt–Ni and PtNi–Au layers; (c) EDX and (d) XPS spectra of as-deposited Pt–Ni and PtNi–Au layers; (e) Comparison of the PtNi–Au alloy composition calculated using EDX, XPS, and SRPES techniques; (f) HRTEM cross-section view and STEM–EDX mapping of the as-deposited PtNiAu15 catalyst.

Despite multiple studies, direct evidence illustrating the detailed composition distribution for such complicated multielement alloys remains obscure. Thus, the understanding of the key factors determining optimization of catalyst design to effectively address both cost-efficiency and stability is still unclear. This work addresses this issue by employing a systematic comparative analysis of the composition–activity–stability correlation of ternary PtNi–Au ORR catalysts using a large portfolio of complementary characterization techniques and electrochemistry supported by density functional theory calculations. Model alloys were prepared via magnetron co-sputtering, utilizing three individual targets as schematically illustrated in Figure 1a, allowing precise adjustment of the alloy composition. The Pt to Ni atomic ratio was maintained at approximately 50:50 for all samples, while Au concentration was progressively increased from 0 to 3, 7, and 15 at % (denoted as PtNi, PtNiAu3, PtNiAu7, and PtNiAu15, respectively).

## 2 Materials and Methods

### 2.1 Sample Preparation

Magnetron co-sputtering was used to deposit PtNi–Au ternary alloys on glassy carbon (GC) substrates (Alfa Aesar) by simultaneously operating three circular TORUS magnetrons (Kurt J. Lesker) positioned at a 45° angle to the substrate. Each magnetron was equipped with 2″ targets of Pt (99.99%, Safina), Ni (99.99%, Kurt J. Lesker), and Au (99.99%, Kurt J. Lesker). Sputtering was conducted in a 0.5 Pa argon atmosphere using the DC mode, with the power on the corresponding magnetrons adjusted to achieve the desired compositions. Monometallic Pt and Au reference layers were deposited by operating only the corresponding magnetrons for each metal. The nominal thickness for all examined layers was standardized to be around 10 nm.

### 2.2 Sample Characterization

#### 2.2.1 Scanning Electron Microscopy

The morphology of the as-deposited layers was analyzed using a Mira 3 (Tescan) microscope operating at a primary electron energy set at 30 keV.

#### 2.2.2 Energy-Dispersive X-ray Spectroscopy

The bulk composition of the as-deposited layers was examined by energy-dispersive X-ray spectroscopy (EDX) using an XFlash detector (Bruker) integrated into the scanning electron microscope.

#### 2.2.3 Transmission Electron Microscopy

Transmission electron microscopy (TEM) measurements were performed using a JEOL-2200FS microscope operated at 200 kV. Cross-sectional lamella was prepared using a dual-beam LYRA (Tescan) microscope. EDX elemental mapping was performed by using a JED-2300 (JEOL) energy-dispersive X-ray analyzer.

#### 2.2.4 Atomic Force Microscopy

The topography of the as-deposited layers was analyzed by using a MultiMode 8 atomic force microscope (Bruker). The measurements were performed in tapping mode under ambient conditions by using SCANASYST-AIR probes (Bruker) with a nominal tip radius of 2 nm. Acquired images were processed using NanoScope 1.9 software (Bruker).

#### 2.2.5 Photoelectron Spectroscopy

Conventional X-ray photoelectron spectroscopy (XPS) was performed by using an EnviroESCA system (Specs) equipped with a Phoibos hemispherical electron analyzer and a monochromatic Al Kα X-ray source (1486.6 eV). The measurements were performed in an ultrahigh vacuum (UHV, 10^–7^ Pa).

#### 2.2.6 High-Resolution Synchrotron Radiation Photoelectron Spectroscopy

The high-resolution synchrotron radiation photoelectron spectroscopy (SRPES) measurements were carried out at the Materials Science Beamline at the Elettra synchrotron in Trieste, Italy. The beamline utilizes a plane grating monochromator to provide narrow band synchrotron light in the 21–1000 eV energy range. The end station features a main UHV chamber with a base pressure of 2 × 10^–8^ Pa, equipped with a Phoibos 150 electron energy analyzer (Specs). To achieve the highest surface sensitivity, an excitation energy of 180 eV was used to measure Pt 4f, Ni 3p, and Au 4f core levels. All photoelectron spectroscopy (PES) spectra were processed using the KolXPD software (Kolibrik.net).

#### 2.2.7 X-ray Diffraction and Reflectivity

The X-ray diffraction (XRD) and X-ray reflectivity (XRR) measurements were carried out by using a SmartLab diffractometer (Rigaku). The diffractometer is equipped with a 9 kW rotating anode X-ray source (Cu Kα radiation, λ = 0.15418 nm), a parabolic multilayer mirror in the primary beam, a set of axial divergence-eliminating Soller slits with an acceptance of 5° in both incident and diffracted beams, and a HighPix-3000 2D hybrid pixel single-photon counting detector. The XRD measurements were performed in the parallel beam glancing angle XRD (GAXRD) geometry. The incidence beam angle was kept constant at 0.6° for the measurements. The XRR curves were modeled using the Parrat formalism.^18^ Reflectivity curves were processed by using custom scripts written in MATLAB. XRD patterns were processed using the MStruct software.^19^

#### 2.2.8 Rotating Disc Electrode

Electrochemical measurements were performed in a half-cell configuration using the rotating disc electrode (RDE) setup (Pine Research) using an SP-150 potentiostat (Bio-Logic). A catalyst layer deposited onto a GC disc (Pine Research, 5 mm diameter, 0.196 cm^2^ surface area) served as a working electrode, while platinum wire (99.99%, Pine Research) was employed as a counter electrode and Ag/AgCl in saturated KCl electrolyte (Pine Research) as a reference electrode. Note that reference and counter electrodes were divided from the main electrochemical cell by a porous frit. Cyclic voltammetry (CV) was performed at room temperature in a N_2_-saturated 0.1 M HClO_4_ electrolyte solution at a 200 mV s^–1^ sweep rate. The ORR activity of the deposited layers was assessed by performing linear sweep voltammetry (LSV) in an O_2_-saturated 0.1 M HClO_4_ electrolyte at a 20 mV s^–1^ scan rate and a 1600 rpm rotation speed. For better comparison with the literature, all potentials were recalculated to a reversible hydrogen electrode (RHE).

#### 2.2.9 Electrochemical Scanning Flow Cell with an Inductively Coupled Plasma Mass Spectrometer

Electrochemical transient dissolution of ^195^Pt, ^60^Ni, and ^197^Au was monitored employing an electrochemical scanning flow cell with inductively coupled plasma mass spectrometry (SFC-ICP-MS) system.^20,21^ A catalyst layer deposited onto the GC plate (Alfa Aesar) served as the working electrode (the working electrode contact area was approximately 0.9 mm^2^). Saturated Ag/AgCl (Metrohm) was employed as a reference electrode and a GC rod (HTW Sigradur G) as a counter electrode. All measurements were performed in a 0.1 M HClO_4_ electrolyte with a flow rate of ca. 200 μL min^–1^ with continuous argon purging. The flow rate varied as the pump tubing aged and was determined carefully for each measurement. Dissolution was measured using a NexION 300 (PerkinElmer) ICP–MS spectrometer via a four-point calibration from Pt, Ni, and Au solutions (Certipur, Merck). The 10 μg L^–1^^187^Re was used as an internal standard for ^195^Pt and ^197^Au, while ^74^Ge was used as an internal standard for ^60^Ni. The total dissolution quantities were determined by integrating the transient dissolution profiles.

### 2.3 Computational Modeling

Spin-polarized density functional theory (DFT) calculations were performed using the periodic plane-wave code VASP.^22,23^ The generalized-gradient exchange–correlation functional by Perdew, Becke, and Ernzerhof (PBE)^24^ was employed in combination with the projector augmented wave (PAW) representation of core electrons.^25^ The cutoff energy for the plane-wave functions was 415 eV. The Brillouin zone was sampled only at the Γ-point. One-electron Kohn–Sham energy levels were smeared by 0.1 eV and the final total energies were extrapolated to zero smearing. During geometry optimization, all atoms were allowed to be displaced locally until forces acting on each atom decreased below 0.2 eV nm^–1^.

Given the granular and rough nanostructure of the films prepared by magnetron sputtering in this work (vide infra), we use nanoparticle models to represent the structure of these systems.^10,26^ The studied 405-atomic truncated-octahedral metal nanoparticles (NPs) with the fcc-type crystal lattices were placed in 3 × 3 × 3 nm^3^ cubic cells, which provided sufficient vacuum space between the periodically repeated NPs to mitigate interactions between them.^27,28^ The 405-atomic NP has 2016 metal–metal bonds and a 204-atomic skin surrounding 201 inner atoms, of which 122 atoms form a subsurface shell and other 60 and 18 atoms form two deeper shells around the central atom. The skin comprises atoms with lower coordination numbers (CNs): 24 corner atoms (CN = 6), 60 edge atoms (CN = 7), 24 atoms in {100} nanofacets (CN = 8), and 96 atoms in {111} nanofacets (CN = 9).

The following findings of previous studies of PtNi and Pt–Au alloy NPs were taken into account for the present calculations of the model PtNi–Au alloys prepared via magnetron sputtering: (i) In PtNi alloys, Pt atoms tend to segregate to the surface and occupy all positions in the NP skin. Ni atoms are preferably located in the core below the skin, where they favorably mix with Pt atoms that did not find empty skin positions, forming Pt–Ni nearest-neighbor atom pairs with heterometallic bonds.^29^ (ii) In Pt–Au alloys, Au atoms are strongly driven to stay on the NP skin, with a preference for less-coordinated positions. After all edge and corner positions are occupied by Au, Au atoms favorably occupy the {111} facet positions. Since the formation of heterometallic Pt–Au bonds is disfavored compared to the homometallic bonds, the NP core exhibits compact Pt fragments inside Au capsules.^10,30,31^

## 3 Results and Discussion

The atomic force microscopy (AFM) and scanning electron microscopy (SEM) images of the as-deposited catalysts are presented in Figures 1b and S1, respectively. All studied samples exhibit a morphology akin to magnetron-sputtered layers characterized by randomly distributed grains with high-angle surface boundaries, serving as a suitable model for the more intricate real supported NP catalysts.^32^ It can also be observed that the addition of up to 15 at % Au to the PtNi layer did not result in any significant morphological variations. The layer thickness and roughness quantified from XRR data remained consistently uniform across all investigated samples, measuring approximately 10 and 1 nm, respectively (Figure S2). The roughness measured from AFM images showed similar uniformity but was slightly lower than that from XRR due to the convolution effect of the AFM tip. This implies that morphology should not markedly influence subsequent measurements of activity and stability, especially dissolution, where the morphology has been shown to play an important role.^33^

EDX and XPS techniques were further employed to determine the composition of the as-deposited PtNi–Au alloys. EDX probes the entire deposited layer, whereas the XPS technique is more surface sensitive, with a probing depth of about 5 nm. The corresponding spectra are presented in Figure 1c,d. The EDX spectra in Figure 1c consist of three main peaks at 7.48, 9.44, and 9.71 keV, assigned to Ni Kα, Pt Lα, and Au Lα, respectively. The relative bulk atomic composition of all analyzed samples, determined from their respective EDX spectra (see Figure 1e), closely aligns with the intended ones, showing only minor deviations.

Figure 1d presents the XPS spectra of the as-deposited PtNi–Au alloys, which exhibit three doublets at about 84, 71, and 65 eV, assigned to Au 4f, Pt 4f, and Ni 3p states, respectively. It is important to mention here that the close proximity of the Au 4f, Pt 4f, and Ni 3p core levels on the photoelectron spectrum provides a distinct advantage of ensuring an identical probing depth for all elements arising from the fact that these core levels are examined under identical photon flux, transmission function, etc.^29,34^Figure 1e summarizes the relative atomic composition of all analyzed alloys, determined from the areas of the respective XPS core levels, considering the photoionization cross sections. The measured compositions displayed only slight discrepancies compared to those obtained from the EDX results, suggesting a high level of consistency with the intended compositions.

To confirm the homogeneity of our sample, we conducted a detailed TEM analysis. Figure 1f presents an HRTEM cross-sectional view of the as-deposited PtNiAu15 sample, clearly revealing the crystalline structure of the material. Additionally, the corresponding STEM–EDX mapping demonstrates a uniform distribution of Pt, Ni, and Au across the entire thickness of the deposited layer, indicating a consistent alloy composition.

**Figure 2:**
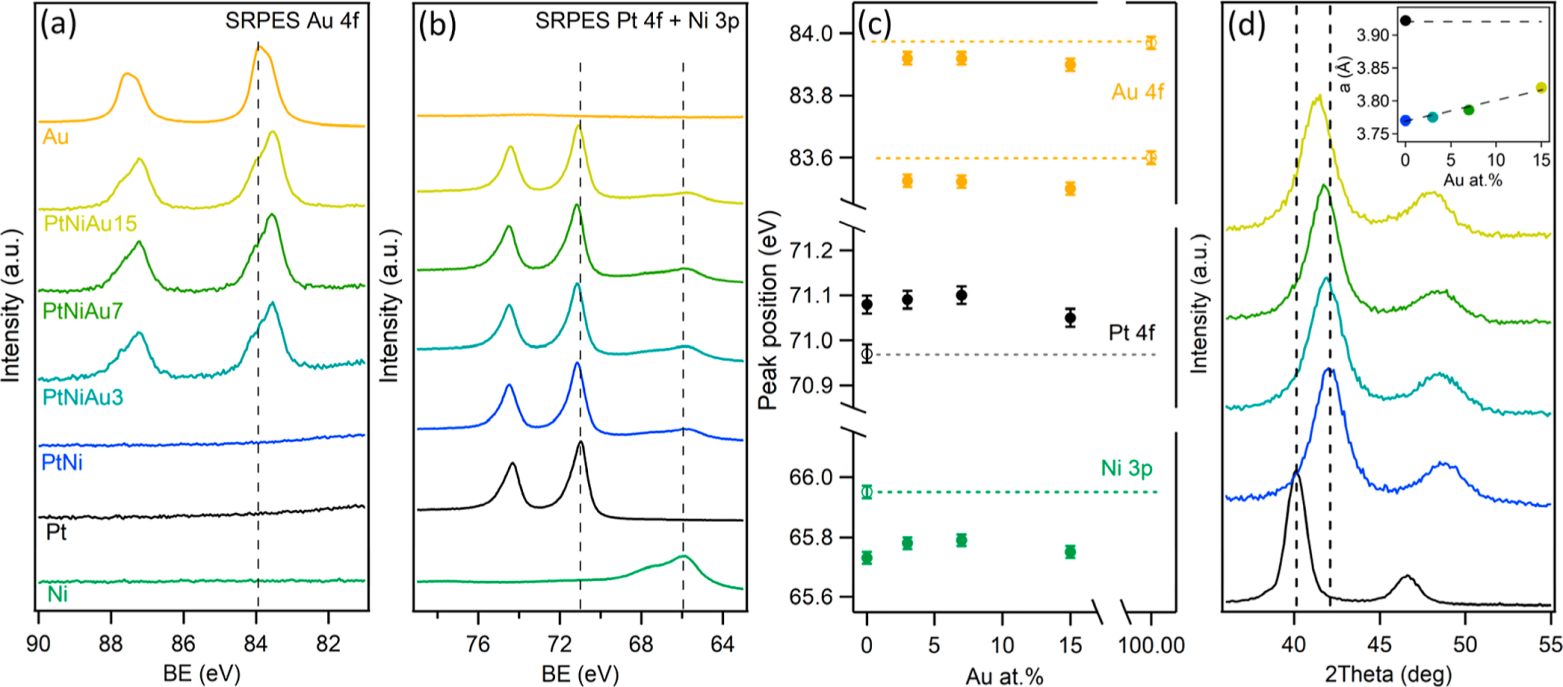
SRPES spectra of (a) Au 4f and (b) Pt 4f + Ni 3p core levels for as-deposited PtNi–Au, PtNi, and monometallic Pt, Ni, and Au layers using 180 eV excitation energy; (c) position of the Au 4f_7/2_, Pt 4f_7/2_, and Ni 3p peaks extracted from the spectra shown in (a) and (b) as a function of Au content (the dotted lines indicate the position of Au 4f_7/2_, Pt 4f_7/2_, and Ni 3p peaks for the monometallic counterparts); and (d) XRD diffractograms of the as-deposited PtNi–Au, Pt–Ni, and monometallic Pt layers (the inset presents lattice parameters, derived from the diffractograms).

The surface-sensitive SRPES, with a probing depth of up to three monolayers, was further utilized to analyze the composition of the outermost layers of the as-deposited PtNi–Au alloys. SRPES spectra of Au 4f and Pt 4f along with Ni 3p recorded for all samples under study, alongside the reference monometallic Pt and Au layers to facilitate the comparison, are depicted in Figure 2a,b, respectively. The relative elemental compositions of PtNi–Au alloys, quantified from their integrated contributions corrected by photoionization cross sections, were Pt_48_Ni_48_Au_4_, Pt_49_Ni_44_Au_7_, Pt_47_Ni_41_Au_12_, and Pt_55_Ni_45_ for the reference layer (Figure 1e). The above compositions align closely with those obtained through EDX and XPS techniques, highlighting that PtNi–Au ternary alloy layers deposited using magnetron co-sputtering possess a relatively homogeneous compositional profile. The only discernible variance is that compositions computed by SRPES consistently exhibit a slightly elevated platinum content, which can be attributed to the thermodynamic tendency of Pt to segregate toward the surface of the PtNi alloy.^29^

The electronic structure of the ternary alloys was then examined in more detail. Figure 2c highlights the position of Pt 4f_7/2_, Au 4f_7/2_, and Ni 3p photoelectron peaks for all samples under study. It can be observed that the Pt 4f and Ni 3p spectra of the reference PtNi layer showed notable shifts compared to their monometallic spectra. Specifically, the Pt 4f_7/2_ upshifted by approximately 0.15 eV from 70.95 eV for monometallic Pt to 71.1 eV for PtNi. Conversely, the Ni 3p downshifted by about 0.3 eV from 66.0 eV for monometallic Ni to 65.7 eV, indicating substantial modification of the electronic structures of Pt and Ni due to their alloying. In contrast to Pt–Au bimetallic systems studied in our previous work,^10^ the position of photoelectron peaks does not exhibit a clear correlative trend with increasing Au content in the PtNi alloy, as seen in Figure 2c. This can be attributed to the more complex interpretation of the photoelectron spectra of the ternary alloys compared to that of a bimetallic alloy. The complexity arises from the superimposed Pt–Au,^10,35^ Pt–Ni,^36,37^ and Au–Ni^38,39^ intermetallic interactions in the ternary alloy system. The impact of these interactions on photoelectrons’ binding energy results in a complex spectrum that is difficult to interpret. Nevertheless, the positions of Pt 4f, Au 4f, and Ni 3p for all PtNi–Au alloys are clearly distinct from those of their monometallic counterparts. In particular, the Pt 4f core level consistently shows a higher binding energy position compared to that of the monometallic Pt. This upshift is usually correlated with a downshift of the Pt d-band center, which reduces the platinum binding affinity to oxygen intermediates, thereby benefiting the ORR.^2,40^

The XRD patterns obtained from the analyzed layers and depicted in Figures 2d and S3 correspond to a single face-centered cubic (fcc) structure (space group *Fm*3̅*m*, no. 225). Notably, the diffractogram of the PtNi alloy is significantly shifted toward higher angles compared to monometallic Pt, which originates from smaller atomic radii of Ni incorporated into the Pt lattice.^41^ Upon the addition of Au to the PtNi alloy, the patterns demonstrate a gradual shift back toward lower angles, suggesting the emergence of tensile strain (with respect to the PtNi alloy) induced by the larger Au atomic radius.^10,42^ The above behavior is quantitatively described by a lattice constant computed from the corresponding patterns (see the inset of Figure 2d). Specifically, for the PtNi alloy, the lattice constant measures 3.77 Å compared to 3.92 Å for monometallic Pt, revealing an approximately 4% lattice contraction relative to Pt. With the stepwise addition of Au, the lattice constant linearly increases in accordance with Vegard’s law, reaching 3.82 Å for the PtNiAu15 sample, which is still approximately 2.5% shorter than that for Pt. This indicates that despite the lattice expansion caused by the addition of Au to the PtNi alloy, compressive strain persists even in the sample with the highest Au concentration compared to monometallic Pt.

Based on the above characterizations, key parameters such as morphology and composition profile were carefully controlled to ensure that only one variable—the amount of Au in the PtNi–Au alloy—was changed at a time. This allows for an accurate assessment of the effect of Au on the activity and stability of the PtNi–Au alloy.

**Figure 3:**
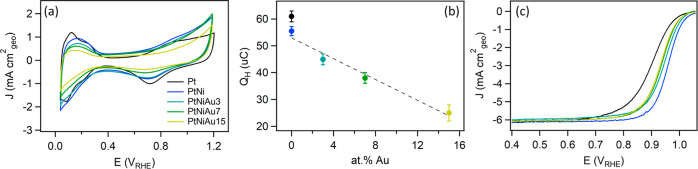
(a) 3^rd^ cyclic voltammograms recorded for the as-deposited PtNi–Au, PtNi, and monometallic Pt layers in N_2_-saturated 0.1 M HClO_4_ solution; (b) H_UPD_ charge calculated from the voltammograms shown in (a) as a function of Au content; and (c) ORR polarization curves of the as-deposited PtNi–Au, PtNi, and monometallic Pt layers recorded in O_2_-saturated 0.1 M HClO_4_ solution at 1600 rpm rotating speed and 20 mV s^–1^ scan rate.

Figure 3a shows the cyclic voltammograms of the PtNi–Au catalysts, along with the reference PtNi layer and monometallic Pt for better comparison, recorded in N_2_-saturated 0.1 M HClO_4_ solution. The voltammogram of the PtNi alloy (blue curve) closely resembles the shape of the cyclic voltammogram of pure Pt (black curve), with the only difference being that it does not exhibit well-resolved peaks in the H_UPD_ region, which corresponds to underpotential adsorption/desorption of hydrogen (H_UPD_) on (110) and (100) Pt step sites exposed to the electrolyte. This can be explained by the formation of a so-called Pt skeleton structure on the surface, that lacks the low-index terminations as a result of Ni leaching from the PtNi surface immediately after contact with the electrolyte solution.^43,44^Figure 3b summarizes the values of the H_UPD_ charge calculated from the corresponding cyclic voltammograms. It can be observed that the *Q*_H_ value for monometallic Pt is comparable to that of the PtNi alloy, which is again related to the dissolution of Ni from the outermost surface of the PtNi alloy. As the concentration of Au was incrementally increased in the PtNi alloy, the *Q*_H_ value began to decrease evidencing blockage of Pt active sites by Au atoms.^10^

Figure 3c shows the ORR polarization curves recorded in an O_2_-saturated 0.1 M HClO_4_ electrolyte for the ternary PtNi–Au catalysts, along with PtNi and Pt monometallic layers to facilitate comparison. In turn, Table 1 summarizes the half-wave potential (*E*_1/2_) and kinetic current (*J*_k_) values at 0.9 V_RHE_ calculated from the corresponding voltammograms. It shows markedly superior ORR activity of the PtNi catalyst compared to monometallic Pt, manifested in a 56 mV upshift of *E*_1/2_ and nearly a 7-fold increase in *J*_k_ at 0.9 V_RHE_, which is well consistent with previous reports.^12,45−47^ The addition of Au and the subsequent increase in its concentration were accompanied by a decrease in the ORR activity. Nevertheless, despite the decrease, the activity of the sample containing the highest amount of Au, namely, PtNiAu15, remained superior to that of pure Pt still showing a 30 mV upshift of *E*_1/2_ and nearly a 3-fold higher value for *J*_k_ at 0.9 V_RHE_. It is also important to highlight that the ORR mechanism is the same for all studied samples, as can be observed from Tafel plots shown in Figure S4.

**Table 1:** Half-Wave Potential *E*_1/2_ and Kinetic Current *J*_k_ at 0.9 V_RHE_ Derived from the ORR Polarization Curves in Figure 3C

	Pt	PtNi	PtNiAu3	PtNiAu7	PtNiAu15
*E*_1/2_ (V_RHE_)	0.900	0.956	0.944	0.935	0.930
*J*_k_ at 0.9 V_RHE_ (mA cm_geo_^–2^)	5.6	40.6	20.6	17.2	15.1

In general, the observed activity decrease with the addition of Au atoms to PtNi could result from two main effects: (i) lowering the ORR activity of the individual exposed active surface Pt(Ni) sites due to the presence of nearby Au atoms (i.e., the ligand effect) or (ii) maintaining the ORR activity of individual surface Pt(Ni) sites, but reducing their number due to covering (hiding) by Au atoms, which tend to replace Pt at low-coordinated skin sites.^10,31^ Considering the known preference of Au to occupy undercoordinated sites of nanoalloys with respect to Pt, it is clear that the number of ORR active Pt sites decreases upon their replacement with inactive Au atoms. However, the influence of nearby Au atoms on the ORR activity of the remaining Pt(Ni) sites, which are still accessible for the ORR intermediates, cannot be excluded.

We conducted DFT calculations on model systems to estimate to what extent the doping of PtNi NPs with Au affects the adsorption energies of relevant ORR intermediates. Specifically, we investigated how the presence of Au atoms in the NP skin impacts the ORR activity of the nearby surface Pt active sites, by comparing the adsorption energies of O and OH on model Pt_303_Ni_100_Au_2_, Pt_305_Ni_100_, and Pt_405_ NPs with ca. 2.2 nm diameter. These adsorption energies are good descriptors for the ORR activity of transition metals, with Pt and PtNi (111) terrace sites appearing near the top of the corresponding activity volcano plot.^48−50^ It is important to mention here that it is crucial to realistically represent the structure of exposed sites in the experimentally studied samples rather than reproduce their overall Pt/Ni composition. For crystallites comprising several thousand atoms (5 nm or larger, as in the present experiments), the number of atoms in the outer surface layer (skin) is close to the number of atoms in the underlying subsurface layer. Therefore, the 1:1 Pt/Ni composition of the experimental samples implies the presence of numerous inner Pt atoms, even when the skin entirely consists of Pt atoms due to their strong preference for these sites. In the case of the modeled 405-atomic NPs, the skin contains 204 atoms. Consequently, 202–203 Pt atoms in the 1:1 Pt/Ni composition would be insufficient to complete the skin, resulting in all inner positions being occupied by Ni atoms. Such a peculiar ordering would be inconsistent with the structures of the experimentally studied samples. On the contrary, the 3:1 Pt/Ni composition in the present calculated models yields a more realistic ordering, with the skin primarily comprising Pt atoms and a Pt/Ni 1:1 composition in the core.

**Figure 4:**
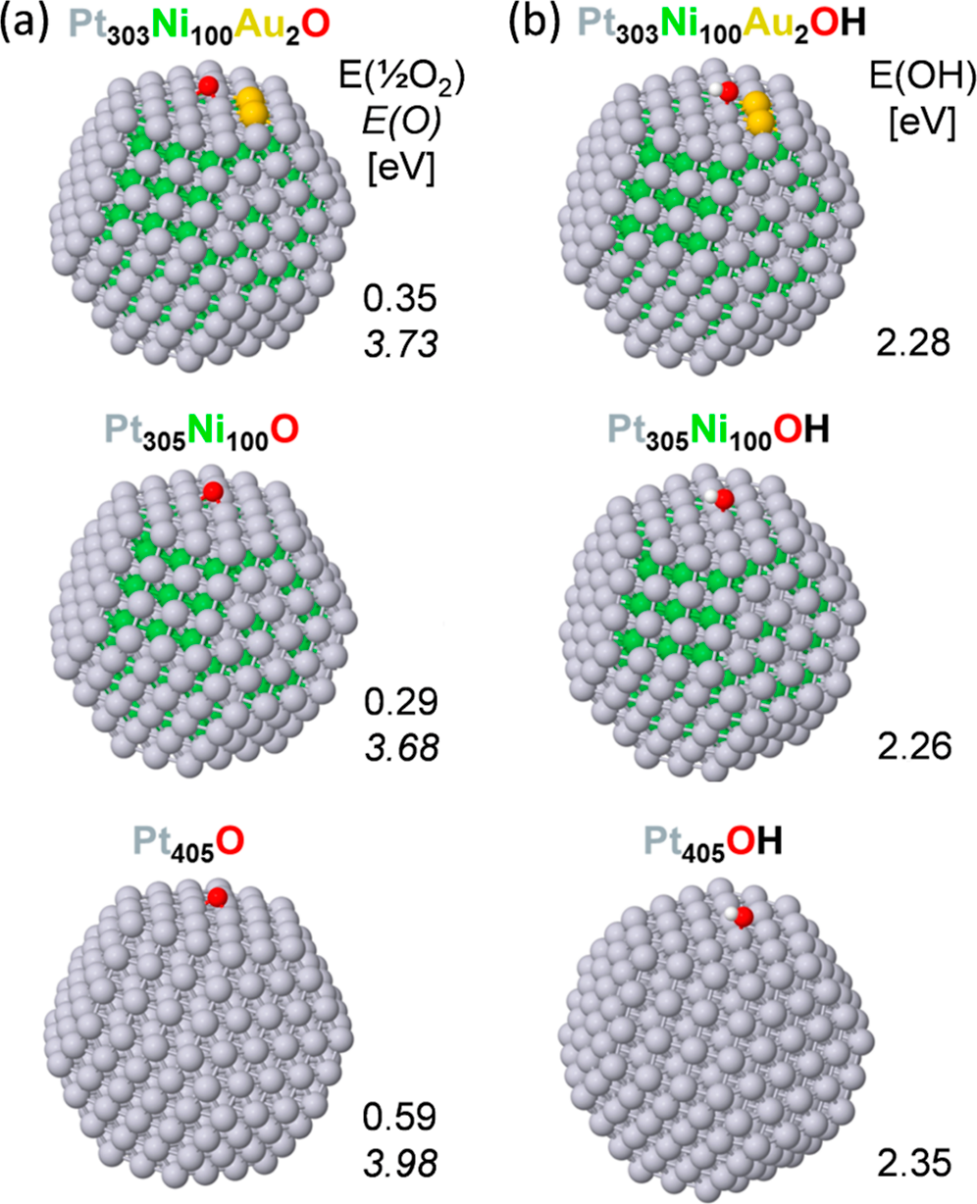
(a) Structure and O adsorption energies on 3-fold hollow PtAu_2_ {111} terrace sites of the Pt_303_Ni_100_Au_2_ NP compared to Pt_3_ sites of the Pt_305_Ni_100_ and Pt_405_ NPs. Adsorption energies *E*_ad_(1/2O_2_) are calculated versus total energies of the NPs without adsorbates and a half of the energy of free O_2_ (adsorption energies *E*_ad_(O) with respect to an O atom are shown in italics). (b) Structure and adsorption energies of on-top OH on platinum {111} terrace sites of the Pt_303_Ni_100_Au_2_ NP compared to those of the Pt_305_Ni_100_ and Pt_405_ NPs. Adsorption energies *E*_ad_(OH) are calculated versus total energies of the NPs without adsorbates and free OH species. Color coding of atoms: Pt—gray, Ni—green, Au—yellow, O—red, and H—white.

The models and results are depicted in Figure 4a,b. These models are consistent with those used to investigate the effects of doping PtNi ORR catalysts with Zr atoms.^51^ Specifically, they involve O adsorption on 3-fold hollow Pt_3_ {111} terrace sites and on-top OH adsorption on platinum {111} terrace sites of the Pt_303_Ni_100_Au_2_ NP compared to the Pt_305_Ni_100_ and Pt_405_ NPs. First of all, the decrease in O adsorption energy from 3.98 eV for Pt to 3.68 eV for PtNi, and of OH adsorption energy from 2.35 eV for Pt to 2.26 eV for PtNi, agrees well with the literature data^45,48−50^ connecting the weakening of bonds formed with the two intermediates to the increased activity of the PtNi alloy (and also measured here by RDE). The introduction of Au atoms to PtNi resulted in just a slight strengthening of the adsorption of O and OH on Pt sites of PtNi–Au due to the ligand effects of the nearby Au atoms. Specifically, the O adsorption energy increased from 3.68 eV for PtNi to 3.73 eV for PtNiAu, and the OH adsorption energy increased from 2.26 eV for PtNi to 2.28 eV for PtNiAu. Nonetheless, the corresponding adsorption energies still remained significantly lower than those observed on pure Pt. This implies that the observed ORR activity decrease of PtNi–Au compared to PtNi could arise not only from a reduced number of active sites due to blocking by Au but also from the somewhat stronger binding of ORR intermediates to the fully accessible Pt active sites near Au.^52,53^

After a thorough examination of the composition–activity correlation of the PtNi–Au alloys, the interplay between composition and stability was screened using in situ SFC-ICP-MS by simultaneously monitoring the online dissolution of Pt, Ni, and Au. The following electrochemical protocol was applied to assess the stability of Pt, Ni, and Au within the investigated alloys. After the sample was contacted with the electrolyte at 0.05 V_RHE_, this potential value was maintained for 5 min. During this period, contact dissolution occurs for most metals, except Au, leading to the emergence of a peak on the spectrograms commonly referred to as a “contact dissolution peak”. In the case of platinum, this peak is typically attributed to the cathodic dissolution that takes place during the reduction of previously formed oxides upon exposure to air.^54^ In contrast, for nickel, this peak arises from a combination of anodic and chemical dissolution processes. After that, two identical CV sweeps from 0.05 to 1 V_RHE_ as upper potential limit were applied. The same protocol was also employed for upper potential limits of 1.2 and 1.5 V_RHE_ on a fresh spot of the same sample to ensure consistent starting conditions.

**Figure 5:**
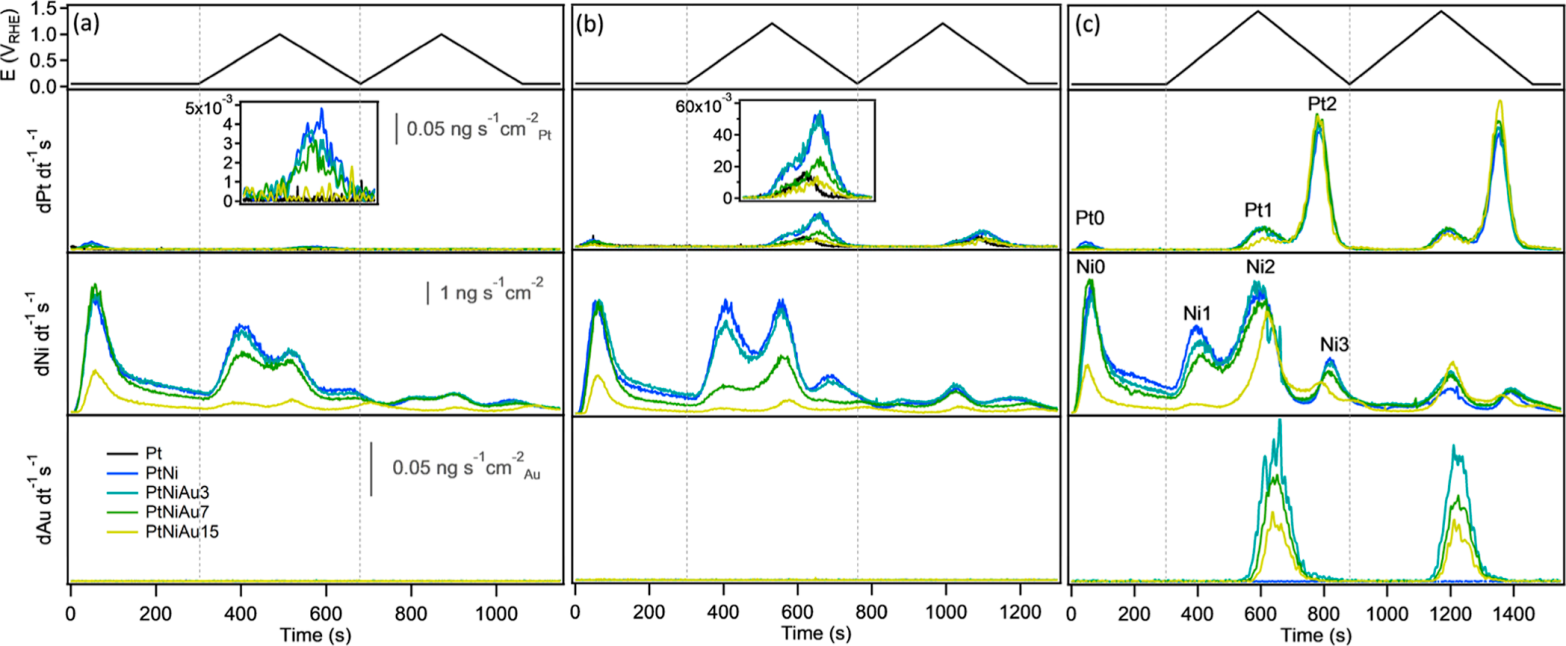
Applied potential protocol together with the corresponding Pt, Ni, and Au dissolution mass spectrograms captured from PtNi–Au, PtNi, and monometallic Pt layers, for (a) 1.0 V_RHE_, (b) 1.2 V_RHE_, and (c) 1.5 V_RHE_ UPLs. The insets in Figures (a) and (b) highlight Pt dissolution during the first cycle at 1.0 and 1.2 V_RHE_ UPLs, respectively.

Figure 5 presents the recorded mass spectrograms of the analyzed elements as a function of time for all investigated PtNi–Au compositions, including reference PtNi and Pt samples, alongside the corresponding potential protocol. To provide a clearer view, Figures S6 and S7 in the Supporting Information include magnified versions of the original plots. All mass spectra of Pt and Au shown here were normalized by the relative presence of the corresponding element on the alloy surface, as determined from the H_UPD_ region (see Figure 3b). In the case of Ni, however, it was not possible to determine its contribution by using CV. Consequently, Ni dissolution mass spectrograms were normalized by the relative amount of Ni in the alloy, as determined by EDX. For clarity, all dissolution profiles are divided by vertical dashed lines into three zones based on the applied potential protocol: contact dissolution (left part), first cycle (middle part), and second cycle (right part).

**Figure 6:**
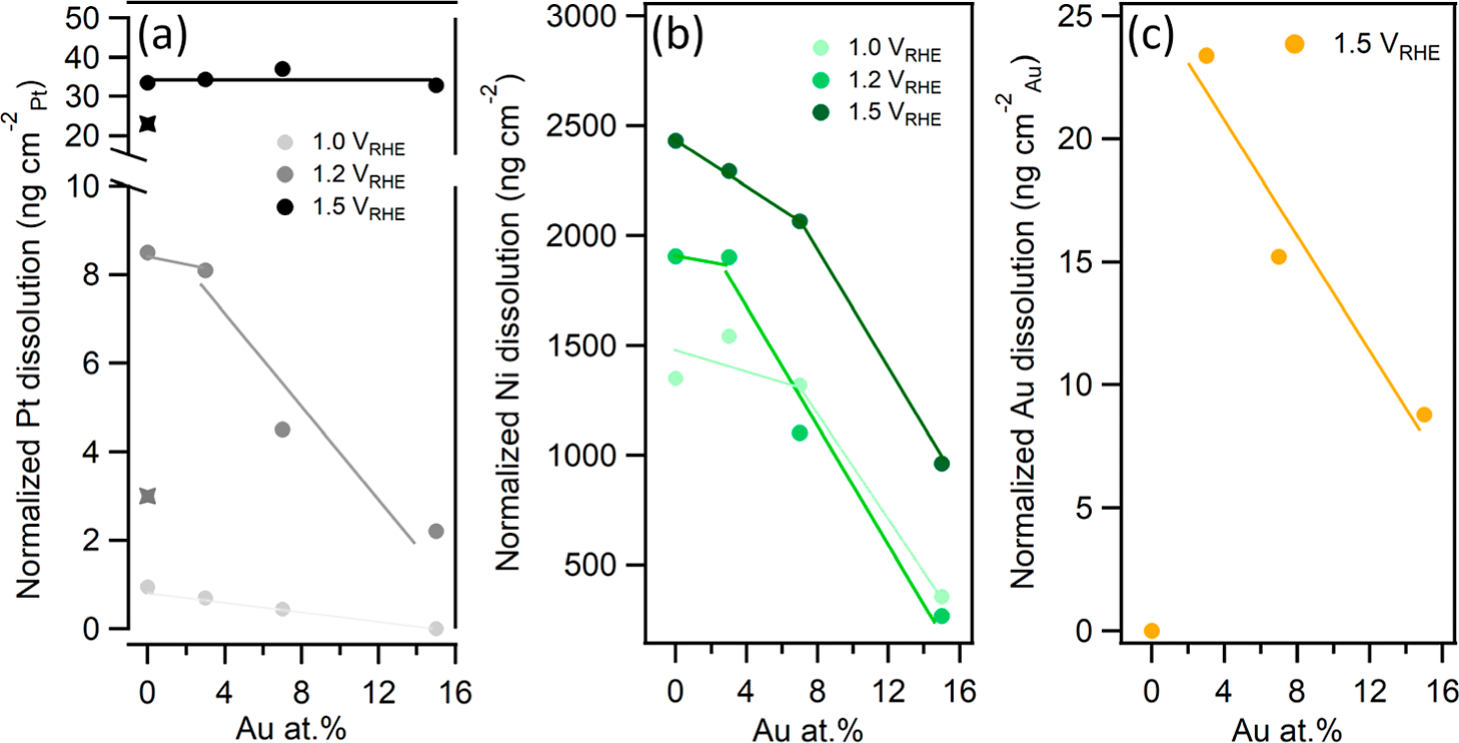
Normalized total dissolution of (a) Pt, (b) Ni, and (c) Au from PtNi–Au and PtNi reference layers calculated by integrating the corresponding mass spectrograms shown in Figure 5 (stars in (a) highlight corresponding Pt dissolution from the monometallic Pt sample). Pt and Au dissolutions are normalized to the relative amount of each element on the alloy surface, as determined from the H_UPD_ region. Ni dissolution is normalized to the relative amount of Ni in the alloy, as determined from EDX spectra.

Typically, at the UPL of 1.0 V_RHE_, it is challenging to detect the dissolution of Pt from monometallic platinum (black curve) as it falls below the detection limit of the ICP–MS (∼3 pg cm^–2^ s^–1^).^55^ Nevertheless, in the case of the PtNi alloy, the increased presence of undercoordinated Pt atoms resulting from Ni leaching from the outermost surface of the PtNi alloy enabled the detection of the Pt dissolution peak (see the blue curve in the inset of Figure 5a). Figure 6a shows the total Pt dissolution values, quantified by integrating the platinum dissolution profiles for all samples and UPLs under study. The first sign of reduced Pt dissolution in the presence of Au was evident already from the analysis of contact dissolution peaks, which are highlighted in Figure S5. Interestingly, with an increasing Au concentration, the intensity of the contact dissolution peak decreased and nearly vanished for the PtNiAu15 sample. This could suggest that the addition of Au may even prevent Pt oxidation already during sample transfer through air, as contact dissolution of noble metals is often linked to the presence of native oxides on their surface.^54^ Indeed, a decrease in the Pt contact dissolution peak has recently been observed in the presence of other noble metals.^56^ The above trend also extends to the Pt dissolution peaks recorded during cycling to 1.0 and 1.2 V_RHE_. Figure 6a shows that at the UPL of 1.0 V_RHE_, Pt dissolution was suppressed compared to the PtNi alloy (0.94 ng cm_Pt_^–2^) by ∼25% (0.7 ng cm_Pt_^–2^) and ∼50% (0.45 ng cm_Pt_^–2^) for PtNiAu3 and PtNiAu7, respectively. More importantly, for the PtNiAu15 sample, the Pt dissolution peak faded, indicating 100% stabilization, at least within the resolution of the ICP–MS device. When the UPL was further increased to 1.2 V_RHE_, no clear decrease in Pt dissolution was observed for PtNiAu3 compared to PtNi. Nevertheless, PtNiAu7 showed nearly a 50% reduction in Pt dissolution, decreasing from 8.5 ng cm_Pt_^–2^ for PtNi to 4.5 ng cm_Pt_^–2^. PtNiAu15 demonstrated an even greater reduction, with Pt dissolution decreasing by approximately 75% compared to PtNi, reaching 2.2 ng cm_Pt_^–2^. Notably, this value is even lower than that recorded for the monometallic Pt sample (3 ng cm_Pt_^–2^). In contrast, no stabilization of Pt dissolution was observed when the UPL was set to 1.5 V_RHE_. The Pt dissolution profiles for all compositions were comparable, averaging around 35 ng cm_Pt_^–2^, which is significantly higher than that of monometallic Pt (23 ng cm_Pt_^–2^). This phenomenon could be attributed to Au dissolution, which occurs solely at this UPL (Figures 5c and 6c). This dissolution may negate the beneficial properties of Au, thereby destabilizing Pt on the surface of the PtNi–Au alloy. Although we did not observe this effect in the case of Pt–Au alloys,^10^ the presence of Ni and numerous defects formed upon its dissolution might contribute to this destabilization. For example, similar results were observed by Gatalo et al., where Pt dissolution in gold-doped PtCu alloys was comparable to the nondoped analogue during a slow scan from 0.05 to 1.4 V_RHE_.^15^

**Figure 7:**
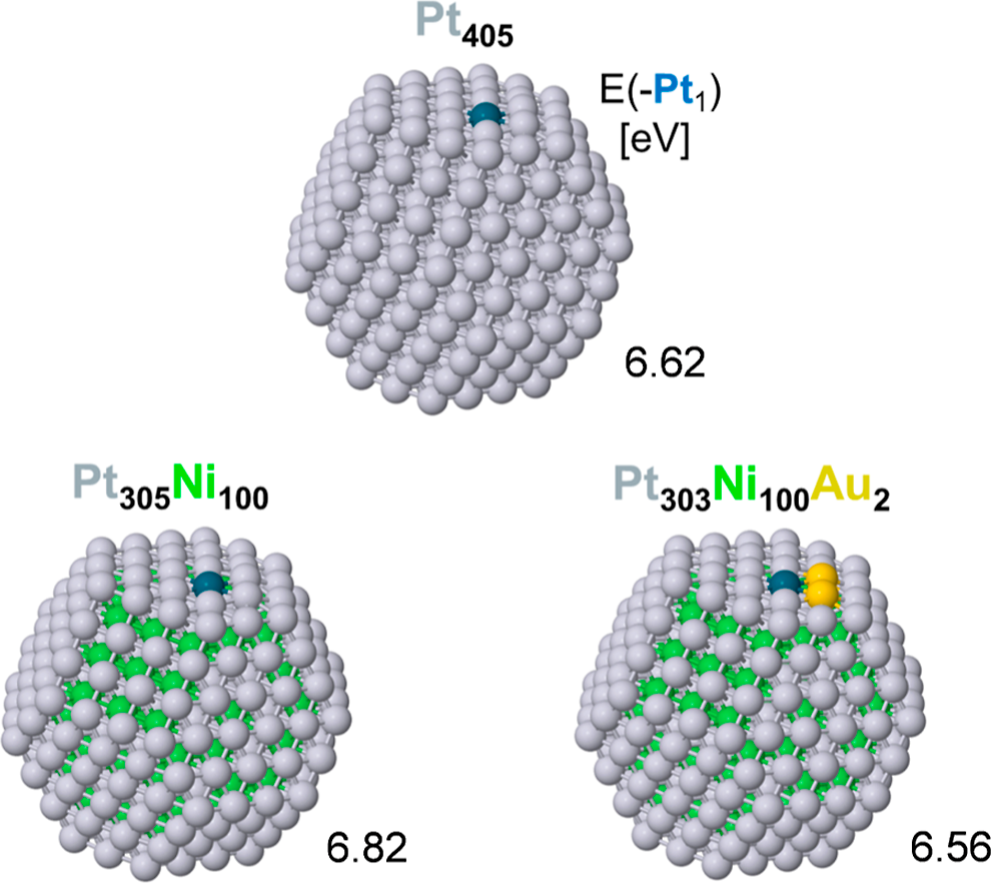
Binding energy of a Pt atom marked in blue on a {111} nanofacet of the Pt_303_Ni_100_Au_2_ NP nearest to two Au atoms compared to binding energies of Pt atoms in the same site of the Pt_305_Ni_100_ and Pt_405_ NPs. Color coding of atoms as in Figure 4.

We further examined the effects of the Au atoms on the stability of nearby Pt atoms in the skin with respect to the detachment of the latter for the Pt_303_Ni_100_Au_2_ NP, comparing them to those of the Pt_305_Ni_100_ and Pt_405_ NPs. The model, which includes two nearest-neighboring Au atoms substituting two surface Pt atoms next to the detached Pt atom, along with the corresponding results, is depicted in Figure 7. These results indicate that the ligand effect of Au atoms does not stabilize near terrace Pt atoms against leaching. Hence, the experimentally observed stabilization of Pt atoms against leaching could be assigned to the geometric hindrance by Au atoms of more prone to leaching Pt substituting them in the less-stable low-coordinated positions, as previously suggested.^9,52^

Overall, the above results are consistent with our previous study of Pt–Au alloys containing 5, 10, and 20 at % Au, where Au atoms were found to be located at coordinatively unsaturated sites, such as corners or edges of the Pt nanoparticles and thus improved durability by suppressing unsaturated-site-induced dissolution of the Pt atoms.^10,52,57^ However, the stabilization effect is less pronounced in the present study, despite the overall higher Au content relative to Pt. This discrepancy could be attributed to the presence of Ni atoms on the surface, which upon their inevitable dissolution create multiple defects on the surface of PtNi–Au alloys. Understanding the dissolution of Ni is thus crucial, as it seems to govern the overall degradation of PtNi–Au alloys.

In contrast to that of Pt, the dissolution spectrogram of Ni in Figure 5 is more intricate. It features several peaks: a contact dissolution peak (Ni0), the anodic Ni dissolution peak (Ni1), and two additional peaks, Ni2 and Ni3, which are attributed to Ni dissolution triggered by the anodic and cathodic dissolution of Pt, respectively.^58^ By comparing Ni dissolution profiles in Figure 5, it is evident that Ni dissolution was also reduced with increasing Au concentration at all UPLs. Figure 6b shows the total dissolution of Ni, quantified by integrating the nickel dissolution profiles for all of the samples and UPLs under study. It indicates that 3 and 7 at % of Au had a minor effect on Ni leaching, while 15 at % Au resulted in a significant decrease in detected nickel across the entire investigated UPL range. Specifically, the Ni dissolution for the PtNiAu15 sample was suppressed with respect to the PtNi alloy by approximately 75%, 80%, and 53% at 1.0 V_RHE_, 1.2 V_RHE_, and 1.5 V_RHE_ UPLs, respectively. Moreover, examining the absolute values of Ni dissolution in Figure 6b, along with simple mass calculations for a monolayer of Ni provided in the SI file (see eq S1), we can conclude that Ni dissolution in the PtNiAu15 sample is limited to the outermost 1–3 catalyst layers only. In contrast, the PtNi reference sample without Au exhibited significantly higher levels of Ni dissolution (Figure 6b). These values suggest bulk dissolution rather than a strictly surface-level process.

A similar effect was previously observed for Pt_25–*x*_Au_*x*_Cu_75_ alloys, where a 50% reduction in Cu dissolution (compared to Pt_3_Cu) took place upon cycling to 1.4 V_RHE_. This reduction was ascribed to the enhanced surface diffusion of Au atoms, which allows for more effective prevention of copper dissolution compared to a pure Pt surface.^15^ Recently, Gao et al. also observed a notable decrease in Ni dissolution from the PtNiAu catalyst compared to the PtNi alloy.^14^ The authors attributed this behavior to an increase in the energy barrier for the outward diffusion of bulk Ni atoms in the presence of Au, which rises to 0.57 eV from 0.40 eV calculated without Au. This effectively slows the diffusion of Ni to the alloy surface, consequently reducing its dissolution. However, the authors reported only the total dissolution of Ni after the entire ADT, making it impossible to differentiate between different forms of dissolved Ni. In our study, the use of slow potential scans allowed for well-resolved individual dissolution peaks. A closer examination of the nickel spectrograms in Figure 5, particularly the individual dissolution peaks, corroborates their findings. The behavior of Ni2 and Ni3 peaks is pretty much dictated by Pt1 and Pt2 dissolution peaks as they correspond to the dissolution of Ni exposed to the electrolyte as a result of Pt dissolution. The only Ni dissolution peak that is independent of Pt dissolution is Ni1 which arises from the anodic dissolution of Ni segregated to the surface due to its oxidation.^29,34^ It is evident from the data that regardless of the upper potential limit, Ni1 decreases with increasing Au content. This decrease is even more pronounced than the decrease in the Ni2 and Ni3 peaks. This undoubtedly confirms the impact of Au on the stabilization of Ni. Based on the above discussion, we can conclude that the suppression of Ni dissolution is a complicated process involving the interplay between surface diffusion of Au atoms and increase in the energy barrier of Ni outward diffusion in the presence of Au.

Overall, our results indicate a negative impact of Au on PtNi activity as evidenced by a significantly lower kinetic current density for PtNi–Au alloys compared to the PtNi alloy. In line with our previous study and other reports on binary PtAu catalysts,^9,10^ we confirmed that the activity decrease with increasing Au content is rather attributed to the blockage of active sites by Au, as supported by DFT calculations. Nonetheless, geometric modifications, particularly tensile strain formation with the addition of Au as indicated by the XRD study, should also play a role and should not be completely overlooked. These modifications could further contribute to the reduced activity. Nevertheless, despite the decrease in the ORR activity, the kinetic current density still remained almost three times higher for the PtNiAu15 catalyst than for monometallic Pt.

On the other hand, the incorporation of Au into the PtNi alloy demonstrated a beneficial effect on its stability in terms of the dissolution of both Pt and Ni, which can be attributed to the structure of the PtNi–Au alloys. Once Ni dissolves from the surface of the alloy upon contact with the electrolyte, it generates numerous defects in the Pt surface structure, which could be predominantly filled with highly mobile Au atoms.^15,59^ However, the amount of Au under study may be insufficient to fill all of the defects, especially in the case of PtNiAu3 and PtNiAu7 samples, leaving a significant number of lower-coordinated Pt sites that are more prone to dissolution. This explains the lack of a notable stabilization unless the amount of Au in the alloy reaches 15 at % which corresponds to roughly 35 at % of Au relative to Pt. Indeed, for the PtNiAu15 sample, Pt dissolution was suppressed by 100% with respect to the PtNi alloy at 1.0 V_RHE_ UPL being comparable with the dissolution of Pt from a monometallic Pt sample. At the same time, Ni dissolution was suppressed by 75%. At an elevated potential limit, i.e., 1.2 V_RHE_, nearly 75% reduction in Pt dissolution and 80% reduction in Ni dissolution were recorded with respect to the PtNi alloy. More importantly, the Pt dissolution value for the PtNiAu15 sample was even lower than that for monometallic Pt. Although no stabilization of Pt occurs at a UPL of 1.5 V_RHE_, this upper potential represents a rare condition in fuel cell operation and can be considered to be less critical in practical applications.

One may have concerns about Au segregation to the surface in PtNi–Au alloys. Based on surface energy considerations, Au is indeed generally expected to segregate over Pt.^60^ However, this behavior is not necessarily applicable under operating conditions relevant to ORR catalysts. It is expected that under oxidizing conditions, Au will not segregate to the outermost surface due to the higher affinity of Pt toward oxygen. Indeed, previous studies on Au-based ternary alloys did not observe significant Au segregation during long-term ASTs, which would otherwise lead to a substantial decrease in ECSA and activity.^12,14,61^ Moreover, first-principles calculations suggest that two competing forces take place: the stronger interaction between Pt and surface oxides, which favors Pt on the surface.^61^ The energetic tendency of Au to preferentially reside on the Pt surface in a vacuum is neutralized by its reduced affinity for adsorbing oxidic species. Consequently, during electrochemical operation at potentials high enough to induce the adsorption of oxidic species, Au will remain beneath the Pt surface.

Nowadays, catalysts of Pt alloyed with 3d metals offer significantly higher activity and reduced costs and thus are replacing pure Pt as a state-of-the-art catalyst. Restraining Ni dissolution is thus equally, if not more, important than Pt dissolution for maintaining the catalytic activity of PtNi alloys. Additionally, reducing Ni dissolution can mitigate the previously reported negative effects of dissolved transition metal ions on the transport properties in ionomers and membranes.^62,63^ Additionally, considering the significantly lower cost of Ni, PtNi–Au ternary alloys present a promising option for cost-effective and high-performance ORR catalysts.

## 4 Conclusions

In this study, we prepared model ternary PtNi–Au alloys with varying Au concentrations using magnetron co-sputtering from three individual targets, allowing precise control over the alloy composition. The Pt/Ni atomic ratio was maintained consistently at approximately 50:50 across all samples, while Au content was systematically increased from 0 to 3, 7, and 15 at %. The morphology and composition of the samples were thoroughly characterized using various techniques such as SEM, XPS, SRPES, EDX, and XRD to ensure that the morphology and composition profile were carefully controlled, with the only variable being the amount of Au in the PtNi–Au alloy. This approach allowed for an accurate evaluation of the impact of Au on the activity and stability of the PtNi–Au alloy catalysts.

RDE technique and computational modeling enabled us to obtain a profound understanding of the composition–activity relationship in these alloys. Our results indicated that the ORR activity of PtNi decreases noticeably with the addition and further increase of Au amount. Such a decrease was attributed to the blockage of surface active sites by unactive Au atoms, as suggested by the DFT calculations. Nevertheless, the PtNi–Au alloy with 15 at % Au still exhibited ORR activity approximately three times higher than monometallic platinum.

At the same time, the composition–stability relationship was examined using SFC coupled with ICP–MS by probing Pt and Ni dissolutions, which are key indicators of the ORR catalyst degradation. Our findings revealed that the presence of Au effectively suppresses the dissolution of both Pt and Ni. While alloys with lower Au content showed limited suppression, the PtNi–Au alloy with 15 at % Au demonstrated notably higher stability compared to the undoped PtNi alloy. For this alloy, Pt dissolution was entirely suppressed at 1.0 V_RHE_ UPL. At the same time, Ni dissolution was reduced by 75%. This trend extended to an elevated potential limit of 1.2 V_RHE_, where Pt dissolution was reduced by nearly 75% and Ni dissolution by 80% compared with the PtNi alloy. Importantly, the Pt dissolution rate for the PtNiAu15 alloy was even lower than that observed for monometallic Pt, underscoring the superior stability imparted by Au doping. The suppression of Pt dissolution was attributed to the stabilization of undercoordinated Pt atoms, while the reduction in Ni dissolution was ascribed to a more complex mechanism involving the surface diffusion of Au atoms and an increase in the energy barrier for Ni outward diffusion in the presence of Au.

## Data Availability

The data presented in this study are available at 10.5281/zenodo.13383621.

## Supporting Information Available

SEM images; thickness and roughness calculated from XRR profiles; XRD patterns; Tafel plots; contact dissolution profiles; and calculation of nickel monolayer weight (PDF)
