# Supplementary material for: Balancing Activity and Stability through Compositional Engineering of Ternary PtNi–Au Alloy ORR Catalysts
Source: ACS Catal. 2024 Dec 16;15(1):234–45. doi: 10.1021/acscatal.4c05269 (PMC11705540; doi:10.1021/acscatal.4c05269)
Supplement: Supplementary file 1 — cs4c05269_si_001.pdf [file cs4c05269_si_001.pdf]

Supporting Information

Balancing Activity and Stability through Compositional Engineering of Ternary PtNi–Au Alloy ORR Catalysts

Xianxian Xie[†], *Valentín Briega-Martos*[‡], *Pere Alemany*[§], *Athira Lekshmi Mohandas Sandhya*[†], *Tomáš Skála*[†], *Miquel Gamón Rodríguez*[†], *Jaroslava Nováková*[†], *Milan Dopita*[‖], *Michael Vorochta*[†], *Albert Bruix*[§], *Serhiy Cherevko*[‡,*], *Konstantin M. Neyman*[§,#,*], *Iva Matolínová*[†], *Ivan Khalakhan*[†,*]

[†] Department of Surface and Plasma Science, Faculty of Mathematics and Physics, Charles University, V Holešovičkách 2, 180 00 Prague 8, Czech Republic

[‡] Forschungszentrum Jülich GmbH, Helmholtz Institute Erlangen-Nürnberg for Renewable Energy (IET-2), Cauerstr. 1, 91058 Erlangen, Germany

[§] Departament de Ciència de Materials i Química Física and Institut de Quimica Teòrica i Computacional (IQTCUB), Universitat de Barcelona, c/ Martí i Franquès 1, 08028 Barcelona, Spain

[‖] Department of Condensed Matter Physics, Faculty of Mathematics and Physics, Charles University, 12116 Prague 2, Czech Republic

[#] ICREA (Institució Catalana de Recerca i Estudis Avançats), Pg. Lluís Companys 23, 08010 Barcelona, Spain

*Corresponding authors
Email: s.cherevko@fz-juelich.de, konstantin.neyman@icrea.cat, ivan.khalakhan@mff.cuni.cz

Supplementary figures

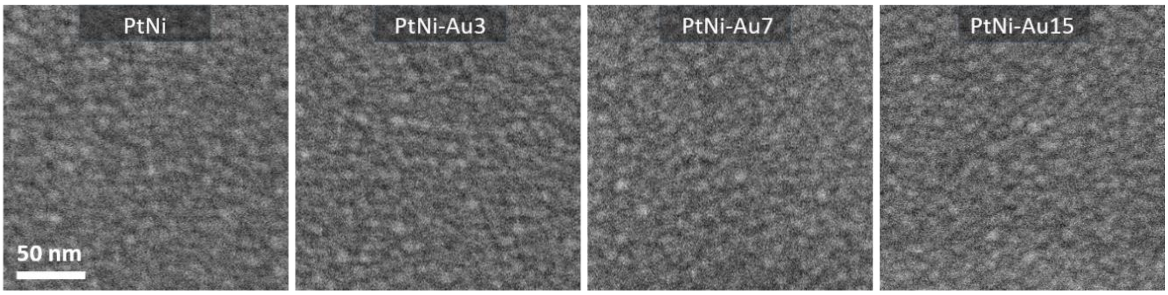

Figure S1. SEM images of the as-deposited Pt–Ni and PtNi–Au alloy catalysts

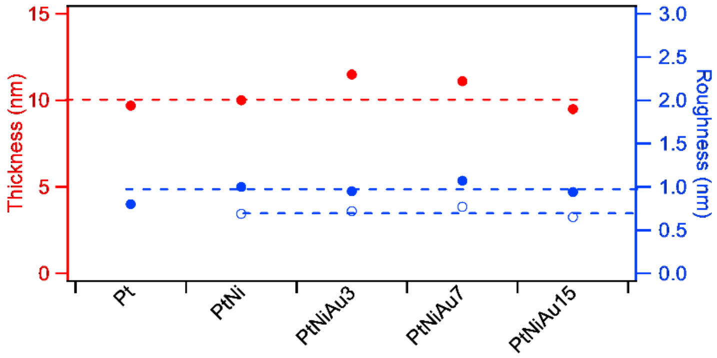

Figure S2. Thickness (left axis, red) and roughness (right axis, blue) values calculated from XRR and AFM. XRR-derived thickness and roughness are shown as filled circles, while AFM-derived roughness values are represented by empty circles.

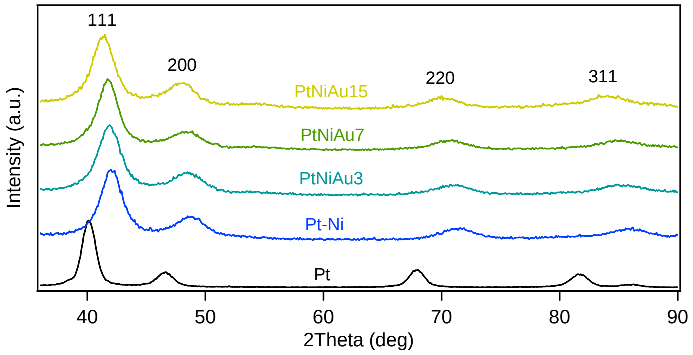

Figure S3. XRD patterns of the as-deposited PtNi–Au, Pt-Ni and monometallic Pt layers

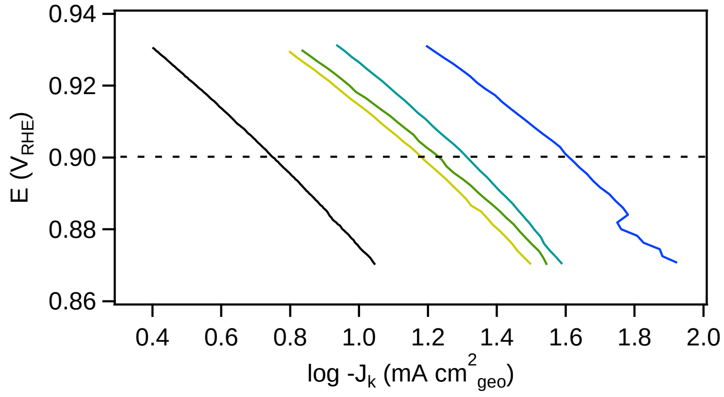

Figure S4. Tafel plots derived from the ORR polarization curves shown in Figure 3c in the vicinity of 0.9 V_{RHE}.

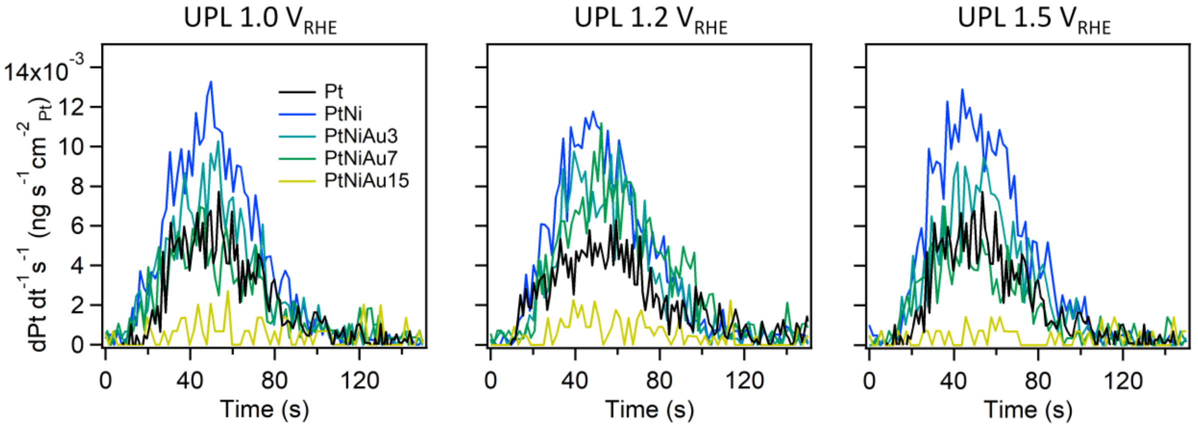

Figure S5. Contact dissolution peak of Pt recorded prior to cycling to different UPLs.

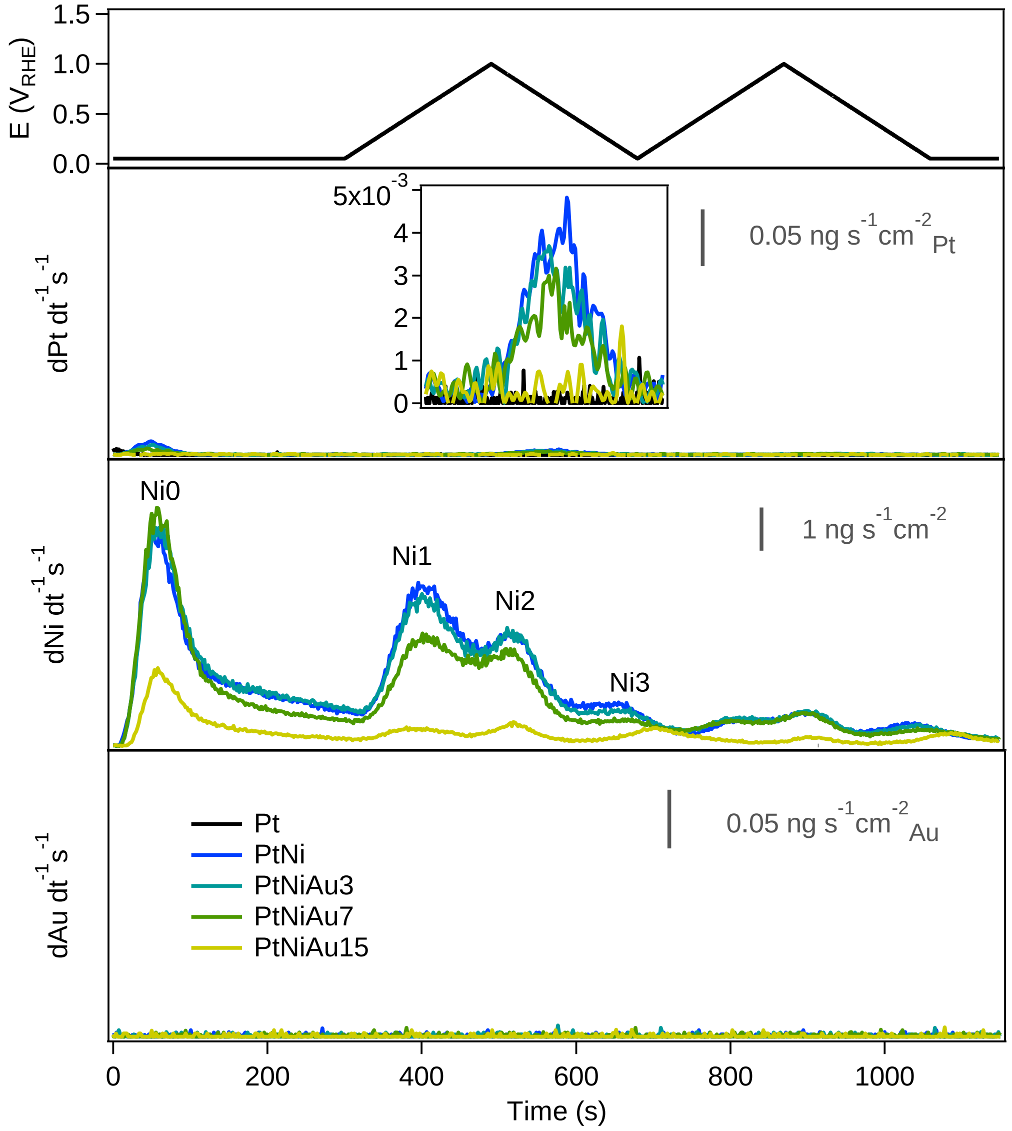

Figure S6. The applied potential protocol along with representative Pt, Ni, and Au dissolution mass-spectrograms taken from PtNi–Au alloy catalysts, as well as the reference PtNi and monometallic Pt electrodes, for 1.0 V_{RHE} UPL. The inset highlights Pt dissolution during the first cycle.

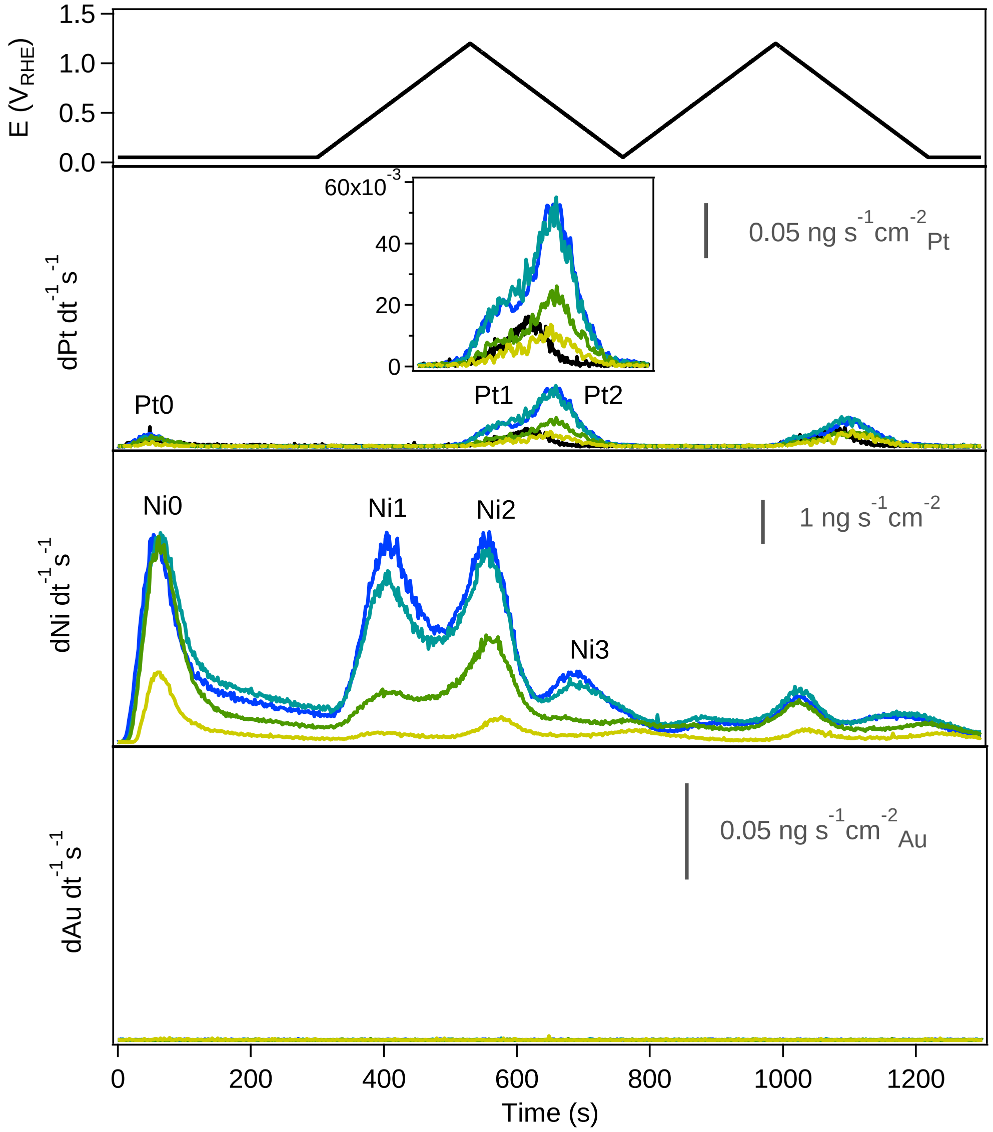

Figure S7. *The applied potential protocol along with representative Pt, Ni, and Au dissolution mass-spectrograms taken from PtNi–Au alloy catalysts, as well as the reference PtNi and monometallic Pt electrodes, for 1.2 V_{RHE} UPL. The inset highlights Pt dissolution during the first cycle.*

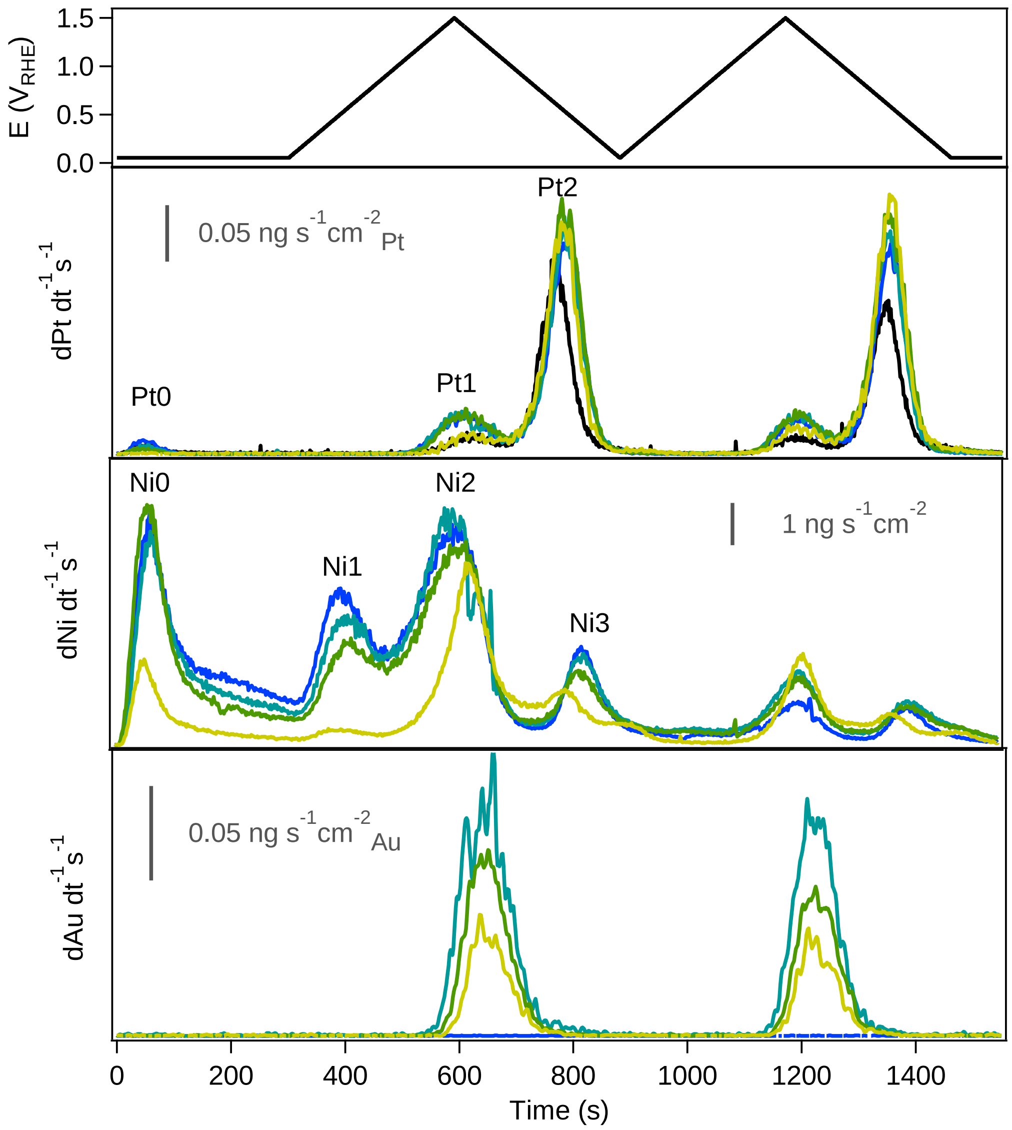

Figure S8. *The applied potential protocol along with representative Pt, Ni, and Au dissolution mass-spectrograms taken from PtNi–Au alloy catalysts, as well as the reference PtNi and monometallic Pt electrodes, for 1.5* V_{RHE} *UPL.*

Calculation of Nickel Monolayer Weight

Regarding leaching from the surface or bulk, we can easily estimate this from ICP-MS results. The weight of a monolayer of Ni per cm^2 can be calculated using the atomic mass of Ni (58.69 $g{\cdot}mol^{-1}$), Avogadro's number (6.022×10^{23} $atoms{\cdot}mol^{-1}$) and surface atomic density of Ni (For close-packed (111) planes of face-centered cubic (FCC) nickel, the atomic density is approximately 1.88×10^{15} atoms/cm^2, considering the area of the unit cell as $S = (\sqrt{3}/2)\times d^2$, and being the atomic diameter for d = Ni 0.248 nm). Then

$$m_{Ni}{\cdot}cm^{-2} = \frac{58.69\ g/mol}{6.022 \times 10^{23}\ atoms/mol}\ 1.88 \times 10^{15}\ atoms{\cdot}cm^{-2} = 185\ ng{\cdot}cm^{-2} \qquad \text{(Eq. S1)}$$

One needs to take into account that:

- Even though the samples contain 40-50 % Ni composition, it is expected that in air, the entire outermost layer of the catalyst would consist of Ni due to its higher affinity for oxygen.[1]
- The calculated value of 185 $ng{\cdot}cm^{-2}$ is based on the assumption of an ideally flat surface. For rough samples, like those in this study (see AFM images in Figure 1b), it is reasonable to expect a higher value.

References

(1) Khalakhan, I.; Vega, L.; Vorokhta, M.; Skála, T.; Viñes, F.; Yakovlev, Y. V.; Neyman, K. M.; Matolínová, I. Irreversible Structural Dynamics on the Surface of Bimetallic PtNi Alloy Catalyst under Alternating Oxidizing and Reducing Environments. *Appl. Catal. B Environ.* **2020**, *264*, 118476.
